# Cluster of Dominant Species and Grazing Jointly Influence the Soil Nitrogen and Phosphorus Cycling in Alpine Grasslands

**DOI:** 10.3390/microorganisms13122736

**Published:** 2025-11-30

**Authors:** Wei Xu, Na Li, Wenting Liu, Weidong Lv, Mengqi Li, Haiming Ji, Yuzhen Liu, Xiaoxia Yang, Quanmin Dong

**Affiliations:** 1 Academy of Animal Science and Veterinary Medicine, Qinghai University, Xining 810016, China; xuweideyouxiang7@163.com (W.X.);; 2 Key Laboratory of Alpine Grassland Ecosystem in the Three-River-Source, Qinghai University, Ministry of Education, Xining 810016, China; 3 Qinghai Provincial Key Laboratory of Adaptive Management on Alpine Grassland, Xining 810016, China

**Keywords:** *Carex alatauensis*, *Potentilla acaulis*, cluster, mixed grazing, nitrogen and phosphorus cycling

## Abstract

This study systematically analyzes the multi-layered regulatory mechanisms of grazing on soil nitrogen and phosphorus cycling functions, based on the combined effects of different grazing strategies and plant community spatial patterns in alpine grasslands. A controlled mixed grazing experiment with moderate intensity was conducted on a livestock system adaptive management platform in the region surrounding Qinghai Lake on the Qinghai–Tibet Plateau, China. The experimental treatments included yak-only grazing (YG), Tibetan sheep-only grazing (SG), mixed grazing of yak and Tibetan sheep (MG), and no grazing (CK). The study quantitatively assessed the soil microbial nitrogen and phosphorus cycling functional genes in the rhizosphere of dominant species, including the *Carex alatauensis* and *Potentilla acaulis*, under different grazing intensities. The aim was to explore the effects of grazing strategy and clusters of dominant species on soil nitrogen and phosphorus cycling and their regulatory mechanisms. The results of this study show that, in the nitrogen cycle, grazing led to a decrease in total nitrogen (TN) content and an increase in ammonium nitrogen content in the dominant species communities. The MG treatment significantly enhanced the abundance of key nitrogen metabolism genes, such as *ureC* and *gs*. In the phosphorus cycle, most grazing treatments increased total phosphorus content, but changes in available phosphorus were variable among plant clusters. The MG and SG treatments significantly increased the abundance of functional genes such as *aphA*, *ugpB*, and *phnW*. Compared to the relatively stable soil nitrogen and phosphorus content, the abundance of functional genes exhibited significantly higher variability across different grazing treatments. The clusters of *Potentilla acaulis* maintained nutrient stability by enhancing nitrogen assimilation and phosphorus uptake, while the clusters of *Carex alatauensis* promoted ammonium nitrogen accumulation through a conservative strategy. The results indicate that grazing influences nitrogen and phosphorus availability by altering nutrient input and disturbance modes, while plant clusters optimize cycling through differential regulation of microbial functional genes in the community. Both factors jointly regulate nitrogen and phosphorus cycling in Alpine Grassland soils. Mixed grazing exhibited significant advantages in promoting nitrogen retention, enhancing phosphorus activation, and improving plant-microbe interactions, reflecting a comprehensive facilitation of nutrient cycling stability in alpine grasslands. These findings provide important theoretical insights for nutrient cycling management and sustainable grazing practices in alpine grasslands.

## 1. Introduction

The Qinghai–Tibet Plateau contains the world’s largest and highest alpine grasslands, which play a crucial ecological role in maintaining regional ecosystem stability and regulating nitrogen (N) and phosphorus (P) cycling. The productivity of grassland ecosystems is constrained by the availability of key nutrients such as carbon (C), N, and P. Among these, N is often the limiting nutrient, whereas P plays a central role in plant and microbial metabolism. Within grassland ecosystems, livestock, vegetation, and soil interact and mutually to regulate one another [1,2]. Previous studies have demonstrated that many of the effects of grazing on grassland ecosystems are mediated by alterations in N and P cycling [3], with soil microorganisms serving as the primary mediators of nutrient exchange across the livestock–plant–soil interface [4]. In addition, long-term grazing can modify root exudation and stimulate microbial activity, thereby influencing N and P cycling processes within the root-microbe-soil system [5].

Existing research indicates that microbial functional genes actively participate in soil nutrient cycling processes, supporting soil function and broader soil health [6]. The relationships between soil nutrients and microbial functional genes have been extensively investigated. For example, during ecological succession at glacier forefields, microorganisms can enhance carbon fixation by selecting more efficient metabolic pathways [7]. Environmental variables play key roles in shaping both the diversity and functional potential of soil microbial communities [8]. Grazing also exerts strong influences on the interactions between soil nutrients and microbial functional genes. It can alter N and P cycling processes and the expression of key functional genes by modifying plant biomass, root exudates, and soil physicochemical properties [9]. However, the direction and mechanisms through which different grazing intensities or livestock assemblages affect N and P cycling remain controversial. Several studies have reported that long-term grazing suppresses the abundance of the denitrification genes *nirS* and *nirK* [4], and that grazing can influence phosphorus-transformation–related genes and enzyme activities by altering the structure of the microbial community [10]. Moreover, plants can promote the assembly of rhizosphere microbial communities by regulating root phenotypic traits and the secretion of root exudates [11]. However, a systematic understanding of the combined regulatory effects of different grazing regimes and plant assemblages is still lacking.

During grazing, large herbivores with contrasting body sizes, physiological traits, and foraging strategies exert markedly different influences on grassland vegetation dynamics. For instance, cattle and sheep differ in both body size and feeding strategy: cattle, being large-bodied and bulk grazers, tend to impose broad pressure on tall dominant species, whereas sheep, being smaller and more selective feeders, prefer to graze nutrient-rich species [12]. The complementary effects of their distinct foraging strata and dung–urine deposition patterns may mitigate ecological biases caused by single-species grazing. Thus, mixed grazing is considered a more ecologically sustainable management strategy, capable of optimizing vegetation structure and nutrient redistribution through interspecific complementarity among livestock.

In natural grassland communities, most plant species exhibit spatial aggregation at one or more spatial scales [13]. Variations in soil nutrient indicators can increase local herbaceous plant density and promote species clustering in suitable microhabitats, while external factors such as grazing can exacerbate resource heterogeneity and consequently alter spatial distribution patterns of plant species [14]. Moreover, long-term grazing in grassland ecosystems often gives rise to a “fertility island effect” [15]. The extent of this effect is strongly influenced by vegetation community structure and plant functional traits [16], and different plant species typically form fertility islands of varying intensity [17]. Microorganisms can indirectly modulate the strength of fertility islands by influencing nutrient cycling processes [18]. The diversity and abundance of microorganisms depend on the quantity and quality of plant litter inputs, as well as on the microclimatic conditions and ecological niches created by plant clusters [19,20]. Therefore, we hypothesize that the distinct species composition within clusters dominated by different plant species, coupled with differential grazing pressures from various livestock types, may result in divergent fertility-island effects among plant clusters.

To elucidate the role of microbial functional genes in soil N and P cycling under different grazing regimes and plant cluster types, we conducted field experiments at the long-term multi-species mixed grazing adaptive management platform on the alpine grasslands of the Qinghai–Tibet Plateau. The objective of this study is to systematically investigate how various grazing regimes influence soil microbial N and P cycling functional genes directly or indirectly—through their effects on the spatial distribution of dominant plant species—ultimately driving N and P cycling in alpine grasslands. Accordingly, we proposed the following hypotheses: H1: Different grazing regimes significantly alter the abundance of soil microbial functional genes associated with N and P cycling, as well as the corresponding nutrient contents, with mixed grazing exerting a more sustainable regulatory effect. H2: Microbial functional genes respond more rapidly to grazing disturbance than do soil nutrient changes. H3: The rhizospheric microenvironment of dominant plant clusters plays a critical regulatory role in mediating grazing effects, exhibiting significant interspecific differences.

## 2. Materials and Methods

### 2.1. General Situation of the Study Area

The grazing experimental area is located within the Alpine Grassland–Livestock System Adaptive Management Platform in Xihai Town, Haiyan County, Haibei Prefecture, Qinghai Province, China (36°55′ N, 100°53′ E), at an average elevation of 3050 m. The region has a plateau continental climate characterized by a short, cool, and humid warm season and a long, cold, and dry cold season. The mean annual temperature is approximately 1.5 °C, with annual sunshine ranging from 2.58 × 10^3^ to 2.75 × 10^3^ h. The long-term mean annual precipitation is about 400 mm, with more than 80% occurring between May and September. The study site represents a typical alpine meadow grassland, with alpine meadow soil and a total vegetation cover exceeding 85%. The dominant species are *Carex alatauensis* and *Potentilla acaulis*, while subdominant species include *Carex aridula* and *Stipa purpurea* [21,22,23].

### 2.2. Experimental Design and Sample Collection

The experimental site for this study was a relatively flat grassland with a homogeneous environment, effectively controlling spatial heterogeneity differences across the experimental plots. Since the establishment of the Alpine Grassland-Livestock System Adaptive Management Technology Platform in 2014, continuous grazing experiments have been conducted according to the design. Grazing occurs during the growing season of alpine grassland plants (June to October), with approximately 10 days of grazing each month, while livestock are transferred to adjacent grasslands for grazing during the remaining time. The grazing experiment employed a randomized block design, with all grazing treatments maintained at a moderate intensity, with a stocking rate of approximately 3.86 sheep units/hectare [22,23]. This intensity was determined based on the goal of maintaining 50–55% utilization of forage. Under moderate grazing intensity, the grazing behavior of cattle and sheep does not completely eliminate dominant species individuals. The moderate grazing intensity trial involved four treatments: yak-only grazing (YG), Tibetan sheep-only grazing (SG), yak-Tibetan sheep mixed grazing (MG), and a no grazing control (CK) treatment. As shown in Table 1, each treatment had three replicates, totaling 12 experimental plots, with each grazing plot separated by a 1.20 m-high net fence. No supplementary feeding was provided during grazing, and drinking water was added every two days.

**Figure 1:**
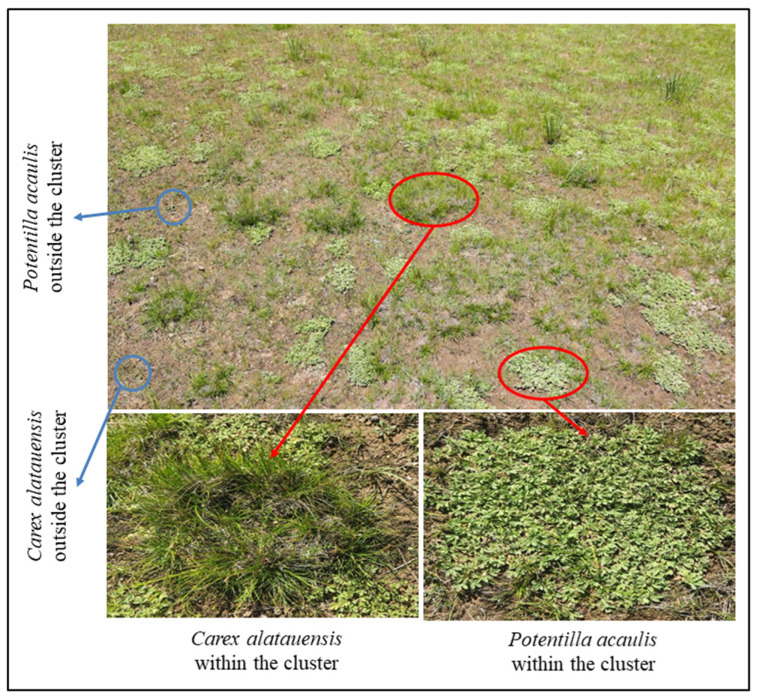
Schematic diagram of sampling site layout. The red circles indicate dominant species exhibiting clustered growth, whereas the blue circles denote dominant species growing individually outside the clusters.

In August 2024, three cluster sites of the dominant species, *Carex alatauensis* and *Potentilla acaulis*, were selected in each plot (Figure 1). Soil samples were collected using a 5 cm diameter soil auger at a depth of 0–5 cm, with three samples collected from each cluster in each site. A total of 9 replicate samples were collected for each treatment within the species clusters. Additionally, three sites outside the dominant species clusters, where the species grew as individual plants (more than 50 cm from the cluster), were selected, and soil samples were collected using the same method. The collected samples were divided into two portions, with 10 g air-dried and 5 g stored at 4 °C in a 2:1 ratio for soil nutrient measurements. Surface soil samples from the same locations were preserved at −80 °C for quantitative analysis of microbial functional genes.

### 2.3. Selection of Soil Microbial Functional Genes and Nutrient Indicators

Given that different plant species may drive distinct microbial functional processes, we selected key N- and P-cycling functional genes from the rhizosphere microenvironments of *Carex alatauensis* and *Potentilla acaulis*. At the *Carex alatauensis*, 10 functional genes involved in N and P cycling were selected [6]. The N-cycling genes included those associated with organic N mineralization (*betB*) and denitrification (*nirD*, *nirS*). The P-cycling genes were associated with organic P mineralization (*phnPP*, *aphA*, *phoN*), P uptake and transport (*ugpC*), P assimilation (*purA*, *purT*), and P reutilization (*adk*). At the *Potentilla acaulis*, 11 functional genes were selected. The N-cycling genes included those involved in organic N mineralization (*ureC*), N assimilation (*gs*, *asnB*, *nirB*), and denitrification (*nirK*, *nosZ*). The P-cycling genes were related to inorganic P solubilization (*ppa*), organic P mineralization (*phnW*), P uptake and transport (*ugpB*, *phnD*), and P reutilization (*dcd*).

To explore the relationships between microbial functional genes and soil element cycling, we analyzed nutrient indicators associated with N and P cycling: (1) N-cycling indicators—total nitrogen (TN), ammonium nitrogen (NH_4_^+^-N), and nitrate nitrogen (NO_3_^−^-N); (2) P-cycling indicators—total phosphorus (TP) and available phosphorus (AP). 

### 2.4. Determination of Soil Microbial Functional Genes

Total DNA was extracted from each sample using the QIAamp^®^ Fast DNA Stool Mini Kit (Qiagen, Hilden, Germany) according to the manufacturer’s instructions. The DNA concentration and purity were determined using a NanoDrop 2000 spectrophotometer (Thermo Fisher Scientific, Waltham, MA, USA), and DNA integrity was verified by agarose gel electrophoresis. Genomic DNA was sheared into fragments using an S220 Focused-ultrasonicator (Covaris, Woburn, MA, USA), and purified with Agencourt AMPure XP beads (Beckman Coulter, Brea, CA, USA). Sequencing libraries were prepared using the TruSeq Nano DNA LT Sample Preparation Kit (Illumina, San Diego, CA, USA) following the manufacturer’s protocol. The libraries were sequenced on an Illumina NovaSeq 6000 platform (Illumina, San Diego, CA, USA), generating 150 bp paired-end reads. Raw sequencing reads in FASTQ format were quality-trimmed and filtered using fastp (v0.20.1) [24]. To remove host contamination, post-filtered paired-end reads were aligned against the host genome using BBMap (v38.93-0), and all mapped reads were discarded. Metagenomic assembly was conducted with MEGAHIT (v1.2.9) [25] using the high-quality reads. Gaps within scaffolds were treated as breakpoints to split them into new contigs (scaftigs), and only scaftigs longer than 500 bp were retained.

Open reading frames (ORFs) were predicted from assembled scaffolds using Prodigal (v2.6.3) [26], and translated into amino acid sequences. Non-redundant gene sets were constructed from all predicted genes using MMseqs2 (v13.45111) with clustering parameters of 95% sequence identity and 90% coverage. The longest sequence within each cluster was retained as the representative gene. Clean reads from each sample were aligned to the non-redundant gene catalog (95% identity threshold) using Salmon (v1.8.0), and gene abundances were quantified accordingly. Taxonomic annotations were assigned based on sequence alignment against the NCBI NR database, and species-level relative abundances were calculated by summing the abundances of genes associated with each species. Functional annotations were performed using DIAMOND (v0.9.7) against the NcycDB [27] and PCycDB v1.1 databases, with an E-value cutoff of 1 × 10^−5^.

### 2.5. Statistical Analysis

All statistical analyses were performed using R software (version 4.2.3). Data normality was assessed using the Kolmogorov–Smirnov test [22]. Descriptive statistics and one-way analysis of variance (ANOVA), followed by Tukey’s post hoc tests, were applied to examine the effects of different grazing treatments on microbial functional gene abundance and N and P cycling indicators. Coefficients of variation (CV) were calculated at both grazing and cluster levels to determine the variability of gene abundance and nutrient indices. To evaluate the effects of grazing, plant clusters, and their interactions on microbial functional gene abundance and nutrient dynamics, linear mixed-effects models were fitted using the glmm.hp package (version 0.1.2) [28]. Multiple linear regression analyses were further conducted to examine the regulatory effects of N- and P-cycling microbial functional genes on nutrient indices. Finally, The Piecewise SEM package (version 2.1.2) was used to construct the Composite Variable Structural Equation Model to further elucidate the primary mechanisms by which grazing methods and dominant species clusters drive changes in soil microbial nitrogen and phosphorus cycling functional genes as well as nitrogen and phosphorus nutrient dynamics. The model was considered acceptable when *p* > 0.05, and a lower Fisher’s C statistic indicated a stronger model explanatory power [29].

## 3. Results

### 3.1. Overall Effects of and Spatial Variations in Grazing on Nitrogen and Phosphorus Cycling Genes and Nutrients

**Figure 2:**
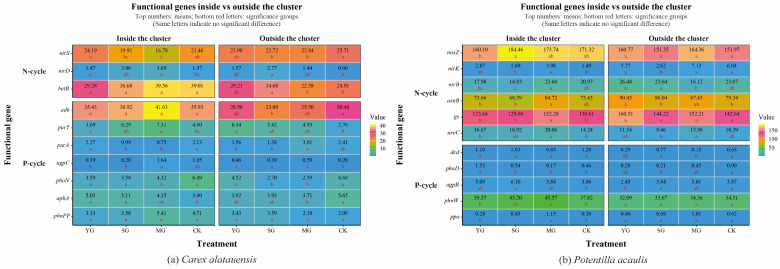
Abundance of Soil Functional Genes in *Carex alatauensis* and *Potentilla acaulis* Clusters under Different Grazing Regimes. Panel (**a**) shows a one-way ANOVA heatmap of the relative abundance of soil microbial functional genes in the rhizosphere, both inside and outside the *Carex alatauensis* cluster. Panel (**b**) shows a one-way ANOVA heatmap of the relative abundance of soil microbial functional genes in the rhizosphere, both inside and outside the *Potentilla acaulis* cluster. YG represents yak-only grazing; SG represents sheep-only grazing; MG represents mixed grazing; CK represents no grazing. The numbers in the figure indicate the mean relative abundance of soil microbial functional genes. Red letters indicate the results of multiple comparisons of the relative abundance of soil microbial functional genes within the same cluster location under different grazing regimes (*p* < 0.05). N-cycle refers to the nitrogen cycling process in the soil, and P-cycle refers to the phosphorus cycling process in the soil.

**Figure 3:**
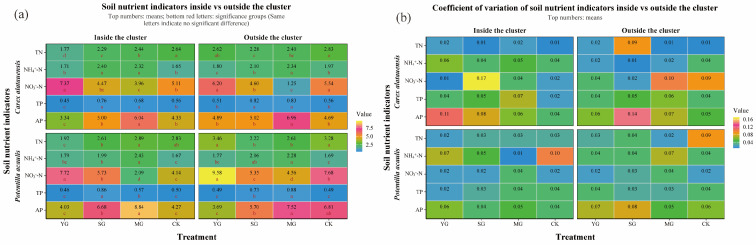
Soil Nitrogen and Phosphorus Indicators and Coefficient of Variation in *Carex alatauensis* and *Potentilla acaulis* Clusters under Different Grazing Regimes. Panel (**a**) shows a one-way ANOVA heatmap of various nitrogen and phosphorus indicators in the rhizosphere both inside and outside the *Carex alatauensis* and *Potentilla acaulis* clusters. The numbers in the figure represent the mean values of soil nitrogen and phosphorus indicators. Red letters indicate the results of multiple comparisons of nitrogen and phosphorus indicator content in the rhizosphere at the same cluster location under different grazing regimes (*p* < 0.05). Units for TC are g kg^−1^, NH_4_^+^-N are mg kg^−1^, NO_3_^−^-N are mg kg^−1^, TP are g kg^−1^, and AP are mg kg^−1^. Panel (**b**) shows a heatmap of the coefficient of variation for various nitrogen and phosphorus indicators in the rhizosphere, both inside and outside the *Carex alatauensis* and *Potentilla acaulis* clusters. Y represents yak-only grazing (YG); S represents sheep-only grazing (SG); MG represents mixed grazing; CK represents no grazing.

**Figure 4:**
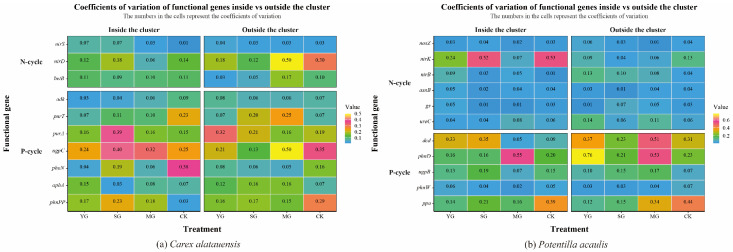
Coefficient of Variation of Soil Functional Genes in *Carex alatauensis* and *Potentilla acaulis* Clusters under Different Grazing Regimes. Panel (**a**) shows a heatmap of the coefficient of variation of soil microbial functional genes in the rhizosphere, both inside and outside the *Carex alatauensis* cluster. Panel (**b**) shows a heatmap of the coefficient of variation of soil microbial functional genes in the rhizosphere, both inside and outside the *Potentilla acaulis* cluster. YG represents yak-only grazing; SG represents sheep-only grazing; MG represents mixed grazing; CK represents no grazing. The numbers in the figure represent the coefficient of variation of soil microbial functional genes. N-cycle refers to the nitrogen cycling process in the soil, and P-cycle refers to the phosphorus cycling process in the soil.

Grazing significantly affected both the abundance of soil N- and P-cycling functional genes and nutrient indices in the two plant clusters, though the direction and magnitude of these effects varied markedly. Overall, grazing enhanced the spatial heterogeneity of soil nutrients and microbial functional genes both within and outside the two species clusters. For N cycling, grazing generally led to a decline in TN content but an increase in NH_4_^+^-N across both specie clusters. In *Carex alatauensis* clusters, MG significantly reduced the abundance of the *nirS* gene, whereas in *Potentilla acaulis* clusters, MG markedly increased the abundance of key genes such as *ureC* and *gs*, suggesting that mixed grazing promotes denitrification and N transformation-related functional activity (Figure 2). The variation in nitrate nitrogen (NO_3_^−^-N) content under different grazing intensities exhibited inconsistent trends, reflecting the complex regulation of N redox processes by grazing (Figure 3). Nitrogen nutrient fluctuations were greater within clusters, while functional gene variability was more pronounced outside the clusters (Figure 4).

Regarding P cycling, grazing significantly altered the abundance of multiple key functional genes and soil P content. In *Carex alatauensis* clusters, SG and MG treatments significantly affected the abundance of *aphA*, *purA*, and *purT*, promoting the accumulation of TP and AP. Within *Potentilla acaulis* clusters, YG significantly increased phnD gene abundance, whereas SG and MG treatments enhanced the expression of *ugpB*, *ppa*, and *phnW* (Figure 2). Most grazing treatments increased TP content, but changes in AP were species-specific—AP generally increased in *Carex alatauensis* clusters but decreased under YG treatment in *Potentilla acaulis* clusters (Figure 3). Overall, the variability in P-cycling indicators was greater than that of N-cycling indicators and was more pronounced outside plant clusters (Figure 4).

### 3.2. Regulatory Mechanisms of Grazing, Species, and Clusters on Functional Genes and Nutrient Indicators

#### 3.2.1. Determinants of Nitrogen and Phosphorus Cycling

**Figure 5:**
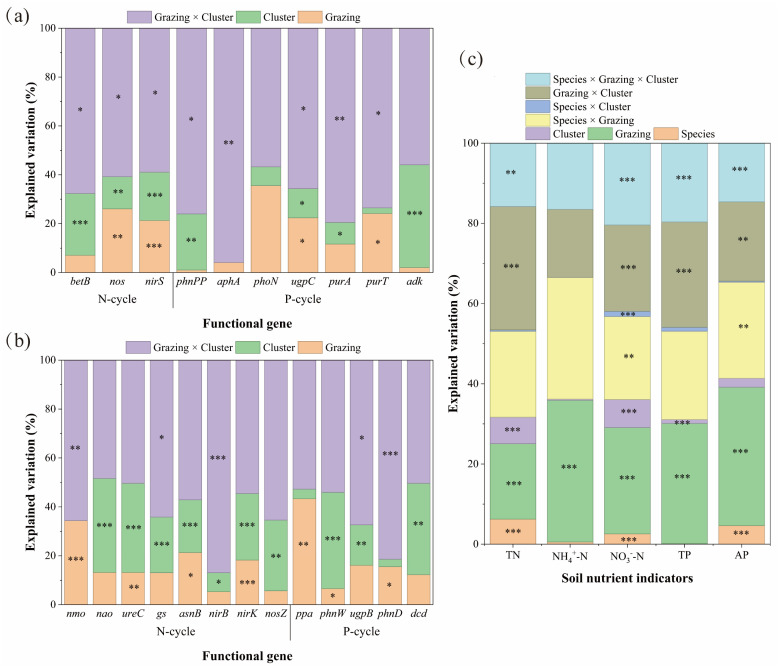
Mixed-Effects Model Analysis of Soil Nutrient Indicators and Functional Gene Abundance of *Carex alatauensis* and *Potentilla acaulis* under Different Grazing Regimes and Cluster Conditions. Panel (**a**) presents the mixed-effects model analysis of grazing and cluster effects on the relative abundance of soil microbial functional genes in the rhizosphere of *Carex alatauensis*. Panel (**b**) presents the mixed-effects model analysis of grazing and cluster effects on the relative abundance of soil microbial functional genes in the rhizosphere of *Potentilla acaulis*. Panel (**c**) shows the mixed-effects model analysis of species, grazing, and cluster effects on soil nitrogen and phosphorus indicators in the rhizosphere. Different colors represent the explanatory rates (%) of various main and interactive effects. N-cycle refers to the nitrogen cycling process in the soil, and P-cycle refers to the phosphorus cycling process in the soil. * *p* < 0.05, ** *p* < 0.01, *** *p* < 0.001.

Results from linear mixed-effects models indicated that the interaction between grazing and plant clusters was the primary driver regulating the abundance of soil N- and P-cycling functional genes. In *Carex alatauensis* clusters, this interaction significantly affected the abundance of N-cycling genes *betB*, *nirD*, and *nirS*, explaining 58.92–67.66% of the total variation, suggesting that grazing and cluster effects jointly dominated the response pattern of N-cycling genes (Figure 5). In *Potentilla acaulis* clusters, the abundance of N-cycling genes was mainly controlled by the main effect of plant clusters, particularly for *ureC*, *asnB*, *nirK*, and *nosZ*, while some genes such as *gs* and *nirB* were more sensitive to interaction effects (Figure 5).

At the nutrient level, variation in TN and NO_3_^−^-N was primarily explained by the grazing and cluster interaction, whereas NH_4_^+^-N was dominated by the main effect of grazing. For P cycling, functional gene abundance in *Carex alatauensis* clusters was also mainly influenced by the interaction term (Figure 5). In *Potentilla acaulis* clusters, *ugpB* and *phnD* were most responsive to the interaction effect, *ppa* was driven primarily by grazing, and *phnW* and *dcd* were influenced mainly by the cluster effect. TP and AP contents were predominantly determined by the main effect of grazing (Figure 5). In summary, the interaction between grazing and plant clusters governed the variation in soil N- and P-cycling functional genes, while the main effect of grazing was dominant in driving changes in soil nutrient concentrations.

#### 3.2.2. Key Processes Influencing Nitrogen and Phosphorus Cycling

**Figure 6:**
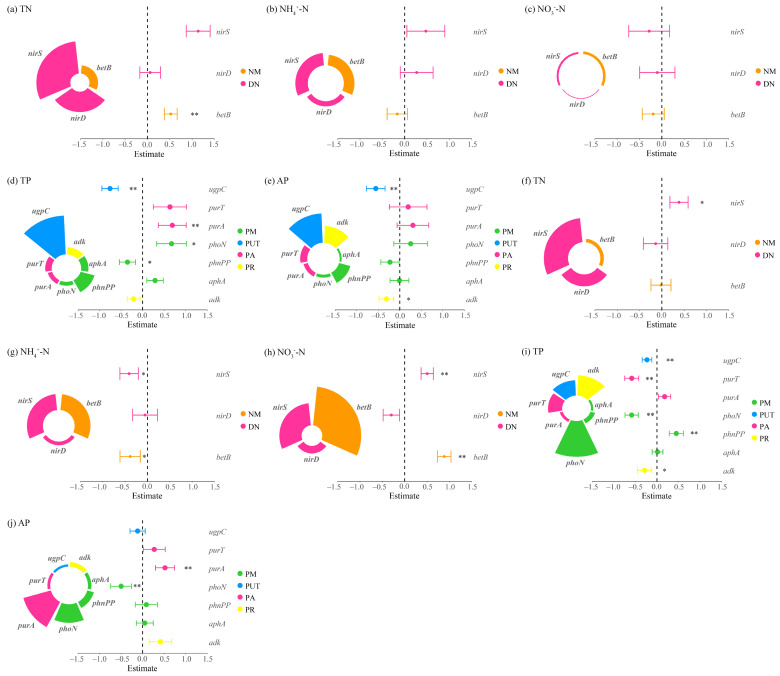
Multiple linear regression analysis of nitrogen and phosphorus cycling microbial functional genes and nitrogen-phosphorus indicators in the rhizosphere soils of *Carex alatauensis*. Panels (**a**–**e**) represent nitrogen and phosphorus contents in rhizosphere soils within *Carex alatauensis* clusters, whereas panels (**f**–**j**) show those in rhizosphere soils of individual plants outside the clusters. Nitrogen (N) and phosphorus (P) cycling processes and their corresponding microbial functional genes are as follows: NM (Nitrogen Mineralization): *betB*; DN (Denitrification): *nirD*, *nirS*; PM (Phosphorus Mineralization): *phnPP*, *aphA*, *phoN*; PUT (Phosphorus Uptake and Transport): *ugpC*; PA (Phosphorus Assimilation): *purA*, *purT*; PR (Phosphorus Recycling): *adk*. Different colors represent functional categories, and the size of each sector indicates the relative contribution of each gene to the corresponding function. Error bars indicate standard errors, and asterisks denote statistical significance (* *p* < 0.05, ** *p* < 0.01). Estimate values represent standardized regression coefficients derived from multiple linear regression models.

Multiple linear regression analyses revealed distinct regulatory mechanisms of N and P cycling between the two clusters of dominant species under grazing conditions. In *Carex alatauensis* clusters, N cycling was primarily controlled by denitrification processes, indicating that denitrification was the key pathway driving N transformation. In soils outside the clusters, organic N mineralization was negatively correlated with NH_4_^+^-N but positively correlated with NO_3_^−^-N, suggesting a directional role of this process in mediating transformations between different N forms. For P cycling, P uptake and transport, along with P reutilization, acted as major negative regulators of both TP and AP, implying that plant uptake may reduce the accumulation of available P (Figure 6).

**Figure 7:**
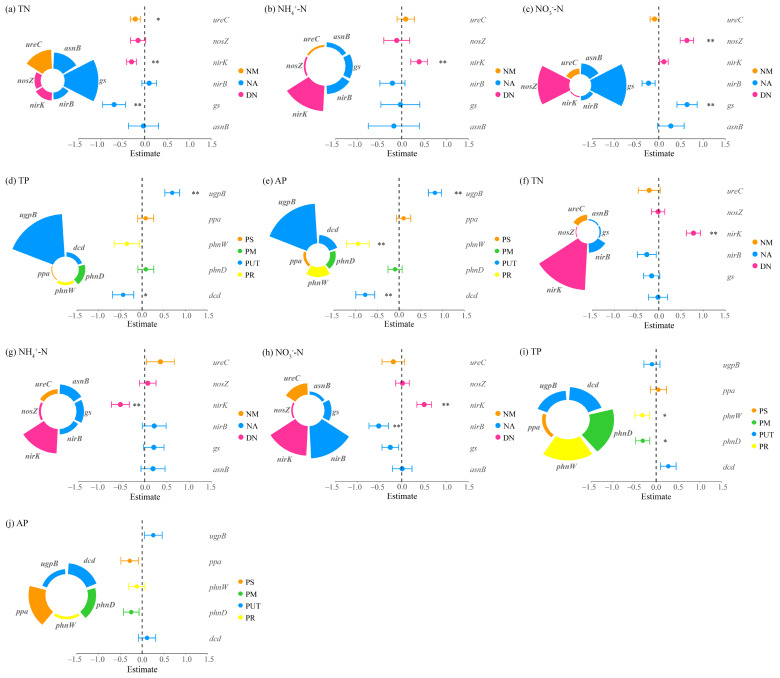
Multiple linear regression analysis of nitrogen and phosphorus cycling microbial functional genes and nitrogen-phosphorus indicators in the rhizosphere soils of *Potentilla acaulis*. Panels (**a**–**e**) represent the nitrogen and phosphorus contents in the rhizosphere soils within *Potentilla acaulis* clusters, while panels (**f**–**j**) show those in the rhizosphere soils of individual plants outside the clusters. The nitrogen (N) and phosphorus (P) cycling processes and their corresponding microbial functional genes are as follows: NM (Nitrogen Mineralization): *ureC*; NA (Nitrogen Assimilation): *gs*, *asnB*, *nirB*; DN (Denitrification): *nirK*, *nosZ*; PS (Phosphorus Solubilization): *ppa*; PM (Phosphorus Mineralization): *phnW*; PUT (Phosphorus Uptake and Transport): *ugpB*, *phnD*; PR (Phosphorus Recycling): *dcd*. Different colors represent various nitrogen and phosphorus cycling processes, and the size of each sector indicates the relative contribution of each gene to the corresponding process. Error bars represent standard errors of the estimates. Asterisks denote significance levels (* *p* < 0.05, ** *p* < 0.01).

In *Potentilla acaulis* clusters, N cycling was dominated by N assimilation processes, which showed a negative correlation with TN and a positive correlation with NO_3_^−^-N, suggesting that this process promotes NO_3_^−^-N accumulation while depleting part of the total N pool. In contrast, denitrification dominated outside the clusters, exhibiting positive relationships with TN and NO_3_^−^-N but a negative relationship with NH_4_^+^-N, indicating opposite trends between reduced and oxidized forms of N. For P cycling, regulation occurred mainly within clusters: P uptake and transport processes significantly affected TP and AP, whereas no significant correlations were detected outside the clusters (Figure 7).

### 3.3. Intrinsic Regulatory Mechanisms of Soil Nitrogen and Phosphorus Cycling Within Dominant Plant Clusters Under Grazing

**Figure 8:**
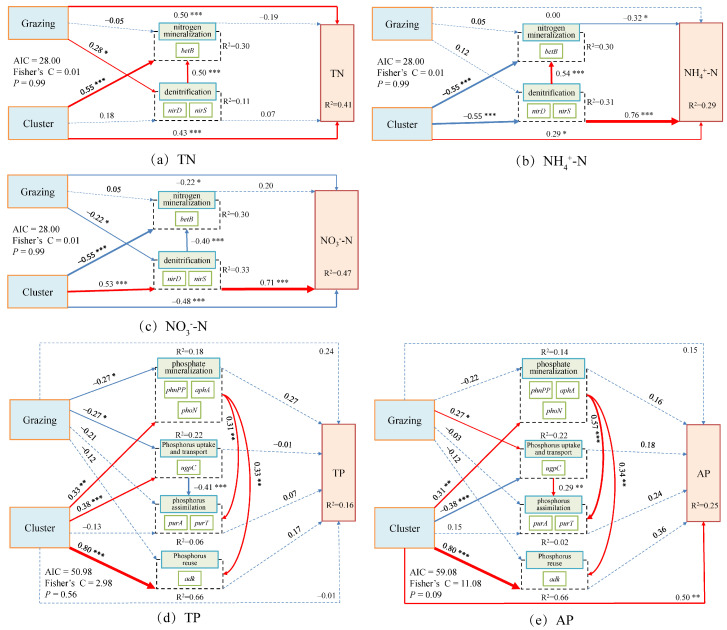
Composite Variable Structural Equation Model of Nitrogen and Phosphorus Cycling Microbial Functional Genes and Nitrogen-Phosphorus Indicators in the Rhizosphere Soils of *Carex alatauensis*. The arrows in the figure represent relationships between variables, with the thickness of the arrows indicating the strength of these relationships. The total standardized effects of the composite variables on soil nutrient indicators are shown as marginal values, while the conditional R^2^ represents the proportion of variance explained by all predictor variables for random effects. Dashed lines represent non-significant relationships, while solid red arrows indicate positive effects, blue solid arrows indicate negative effects, and thicker solid lines represent the magnitude of path coefficients. The significance levels of the predictor variables are indicated as * *p* < 0.05, ** *p* < 0.01, *** *p* < 0.001.

Structural equation modeling (SEM) results revealed that grazing and plant clusters jointly influenced soil N and P cycling processes in both dominant species, though the pathways and directions of these effects differed substantially. In *Carex alatauensis* clusters, grazing and cluster effects on TN and NO_3_^−^-N were opposite in direction, while the cluster exerted both direct and indirect effects on NH_4_^+^-N, highlighting its pivotal role in N transformation. For P cycling, only the cluster showed a significant positive direct effect on AP, whereas both grazing and cluster effects positively influenced P reutilization, indicating that these factors jointly enhanced biological P cycling and reutilization (Figure 8).

**Figure 9:**
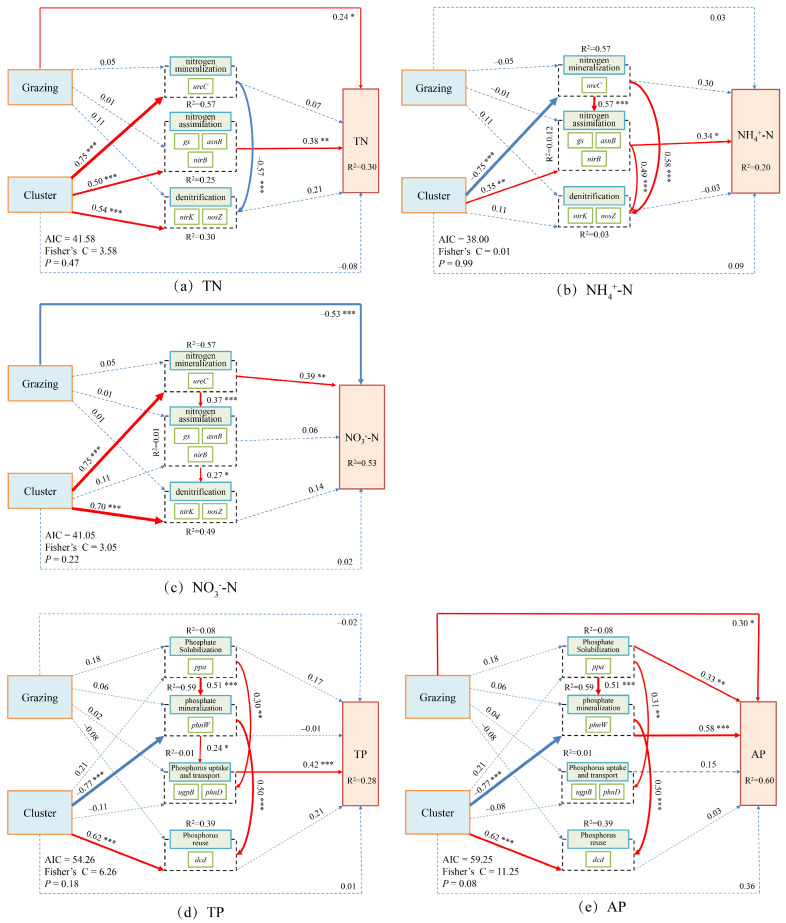
Composite Variable Structural Equation Model of Nitrogen and Phosphorus Cycling Microbial Functional Genes and Nitrogen-Phosphorus Indicators in the Rhizosphere Soils of *Potentilla acaulis*. The arrows represent the relationships among variables, with arrow thickness indicating the strength of these relationships. The total standardized effects of the composite variables on soil nutrient indicators are displayed as marginal values, while the conditional R^2^ represents the proportion of variance explained by all predictor variables for random effects. Dashed lines indicate non-significant relationships; solid red lines indicate positive effects; blue solid lines indicate negative effects; and thicker lines represent stronger path coefficients. The significance levels of the predictor variables are indicated as * *p* < 0.05, ** *p* < 0.01, *** *p* < 0.001. This model illustrates the direct and indirect effects of grazing and plant cluster interactions on soil nitrogen and phosphorus cycling in the rhizosphere of *Potentilla acaulis*. Solid red and blue paths indicate the direction and magnitude of these relationships, reflecting the positive and negative regulatory effects of microbial functional genes and environmental factors on nutrient dynamics in alpine grassland soils.

In *Potentilla acaulis* clusters, grazing exerted significant direct positive and negative effects on TN and NO_3_^−^-N, respectively, whereas the cluster indirectly promoted the accumulation of TN and NO_3_^−^-N through the combined influence of N assimilation and denitrification. Grazing had no significant direct effect on NH_4_^+^-N, suggesting its regulation of N cycling occurred primarily through indirect pathways. Regarding P cycling, grazing significantly increased AP content, while the cluster indirectly enhanced TP and AP accumulation through organic P mineralization and P uptake and transport, with organic P mineralization playing a more prominent role in increasing AP (Figure 9).

## 4. Discussion

### 4.1. Response Mechanisms of Soil N- and P-Cycling Functional Genes and Nutrient Indices to Grazing

#### 4.1.1. Changes in N-Cycling Functional Genes and Nutrient Contents

From the perspective of interactions between livestock assemblages and the spatial configuration of plant clusters, this study reveals multilayered regulatory mechanisms by which grazing modulates soil N cycling in alpine grasslands. Overall, grazing generally decreased TN while promoting the accumulation of NH_4_^+^-N, indicating an accelerated conversion of N from organic to inorganic forms. In *Carex alatauensis* clusters, MG significantly reduced the abundance of the key denitrification gene *nirS*, suggesting suppression of denitrifying microbial activity under this regime. During long-term grazing, the reduction in plant litter, the decrease in soil moisture, and the intensified competitive nutrient uptake by plants collectively lead to a weakening of denitrification, which is consistent with previous findings [4,30,31].

*Potentilla acaulis* clusters exhibited a contrasting response. Under YG, NO_3_^−^-N increased while TN declined, implying that yak grazing may enhance organic N mineralization and nitrification, thereby promoting inorganic N accumulation but weakening biological N retention. By comparison, SG appeared to rely more on N compensation via dung inputs, elevating NH_4_^+^-N while reducing NO_3_^−^-N. Differences in the excretory characteristics of livestock with varying body sizes may regulate the structure of soil nitrogen pools [32]. Under MG, the abundances of *ureC* and *gs*, which are key genes involved in urea hydrolysis and nitrogen assimilation, were significantly increased, indicating that mixed grazing enhanced nitrogen reutilization and internal cycling in the rhizosphere and substantially altered rhizospheric nitrogen processes. Previous studies show that mixed grazing shifts biomass allocation toward belowground compartments [33]. By modifying plant growth conditions, mixed-species grazing can alter the types and amounts of root exudates, thereby reshaping rhizosphere microbial composition and function [34]. In the micro-oxygenated rhizosphere of *Potentilla acaulis*, formed by its complex root system, stronger denitrification can deplete NO_3_^−^-N, producing a distinctive pattern of NH_4_^+^-N accumulation and net NO_3_^−^-N reduction. Taken together, MG exhibits superior ecological regulatory effects on the nitrogen cycle, as it not only reduces nitrogen losses caused by denitrification but also enhances the efficiency of internal nitrogen cycling.

#### 4.1.2. Changes in P-Cycling Functional Genes and Nutrient Contents

Grazing also exerts differential regulation on phosphorus cycling functional genes at different species and cluster locations. In *Carex alatauensis* clusters, MG and SG significantly increased the abundances of P-metabolism genes (*aphA*, *purA*, *purT*), concomitantly promoting TP and AP accumulation. Notably, the concordant upregulation of P-metabolism genes under MG was most pronounced, indicating that complementary livestock effects enhanced P bioavailability [35]. In *Potentilla acaulis* clusters, grazing generally elevated genes involved in organic P transport and mineralization; MG significantly increased *ugpB* and *phnW*, suggesting that this regime facilitates P activation and plant uptake through multiple pathways. In contrast, although YG increased *phnD*, AP declined, implying that stronger trampling or foraging by yaks relative to sheep may constrain P availability [12]. This aligns with Liu’s observation that grazing can increase rhizospheric bacterial abundance and promote P solubilization and mineralization [11]. Gao further demonstrated that plant species produce fertility islands of differing strength in desert ecosystems, with major consequences for P cycling [36]; our findings indicate analogous patterns in alpine grasslands. Overall, MG enhanced P activation and reutilization in both plant clusters—especially elevating P transport and metabolic efficiency in *Potentilla acaulis*—highlighting synergistic gains in nutrient supply and microbial functional regulation consistent with Yang and Liu [33,37].

#### 4.1.3. Variability and Ecological Adaptability of Functional Genes and Soil Nutrients

This study reveals distinct response patterns of soil microbial functional genes and nitrogen–phosphorus nutrient indicators to grazing disturbance. Compared with the relatively stable N and P pools, functional genes—as direct regulators of microbial metabolic processes—exhibit much higher sensitivity to resource fluctuations. Similar patterns have been observed in fertilization experiments [6], indicating that microbial functional regulation can serve as a rapid response mechanism of ecosystems to external disturbances. Meanwhile, plant clustering effects play an important role in maintaining the stability of N and P cycling. *Potentilla anserina* clusters demonstrated greater nutrient stability, which may be attributed to their well-developed root systems and core rhizosphere microbial communities. Phosphorus-cycling indicators were more environmentally sensitive than nitrogen-cycling indicators, likely due to the complex chemical forms of phosphorus and its stronger fixation in soils. Recent advances in global microbial ecology suggest that grassland ecosystems commonly exhibit a pattern of “high sensitivity of microbial functions but relative stability of soil nutrient pools,” meaning that functional gene networks are more easily affected by disturbances than nutrient reservoirs. These findings indicate that alpine grassland microorganisms possess the capacity to rapidly buffer disturbances by modulating key metabolic pathways. They further highlight the importance of microbial functional genes as sensitive indicators for assessing ecosystem stability, underscoring their value in global change research.

### 4.2. Regulatory Mechanisms of Soil N and P Cycling Under Different Grazing Regimes

#### 4.2.1. Interactive Regulation of Grazing and Dominant Plant Clusters

Mixed-effects modeling integrating soil N and P cycling indicates that grazing affects the ecosystem not as an isolated process but through profound interactions with plant-cluster microenvironments, with clear species and element specificity. Plants can adapt to herbivory by altering the chemistry of root exudates to recruit protective, beneficial microbes [38]. Hence, the impacts of grazing on microbial functional genes cannot be ascribed to grazing alone; plant-mediated changes are equally pivotal, and their interactions must be considered. In N cycling, *Carex alatauensis* clusters regulated rhizospheric N-functional gene expression and N-pool dynamics via grazing and cluster interactions; the interplay between root exudates and livestock excreta determined the pathways of N retention versus loss, reflecting active adaptation to grazing disturbance [32,39]. *Potentilla acaulis* clusters exhibited even stronger rhizospheric control, directionally modulating the urea-hydrolysis gene *ureC* to maintain microenvironmental advantages while suppressing neighbors—an ecological strategy underpinning its dominance [40]. For P cycling, the interaction term likewise dominated regulation of *ugpB* and *phnD*, whereas the inorganic P-solubilization gene *ppa* and certain metabolic genes (*phnW*, *dcd*) were primarily driven by grazing or cluster main effects, respectively, underscoring diverse regulatory pathways. Grazing may enlarge plant and litter P pools [41] and accelerate P cycling by altering plant–microbe interactions [35]. By contrast, TP and AP were chiefly determined by the direct effects of grazing, indicating that excreta inputs and trampling impose system-wide influences on the P pool that outweigh microenvironmental heterogeneity [11]. Together, these findings suggest that microbes adapt locally via flexible gene expression, whereas bulk soil nutrient pools respond to grazing-driven resource inputs—highlighting multilevel ecosystem responses to grazing in alpine grasslands.

#### 4.2.2. Differential Regulatory Pathways of N and P Cycling

The results of the study indicate that, during the nitrogen cycling process, different dominant species clusters exhibit significant spatial variations and regulatory pathways. In *Carex alatauensis* clusters, denitrification was significantly negatively correlated with TN, indicating a major pathway of N loss. Changes in organic nitrogen mineralization in the outer soil reflect an increased risk of nitrogen loss as the nitrogen pool diminishes, which is consistent with the findings of Zhou et al. [42]. *Potentilla anserina* clusters exhibited clear spatial heterogeneity: internal variations in total nitrogen and nitrate nitrogen were primarily driven by nitrogen assimilation processes, whereas denitrification dominated in the external zone. These results indicate that this species regulates nitrogen-transformation pathways through rhizosphere microenvironment modification, thereby achieving nitrogen redistribution and utilization. This demonstrates an adaptive nitrogen-cycling strategy of the species under grazing disturbance [39].

In terms of phosphorus cycling, within the dominant species clusters, significant regulation of TP and AP content was observed through phosphorus uptake and transport processes. Under grazing, plants stimulate extracellular enzyme activity and enhance microbial activity by releasing more carbon into the rhizosphere, which ultimately promotes soil phosphorus transformation and nutrient uptake by plants [43]. Outside the *Carex alatauensis* clusters, the total phosphorus content was co-regulated by phosphorus uptake, transport, and recycling processes, while the available phosphorus content was primarily controlled by the phosphorus assimilation process. This suggests that microbes maintain phosphorus stocks and biological fixation, influencing available phosphorus content through uptake and recycling. In contrast, outside the *Potentilla acaulis* clusters, phosphorus content showed no significant correlation with any soil phosphorus cycling processes, possibly indicating a state of “phosphorus equilibrium” or dominance by abiotic factors. This highlights the spatial reshaping effect of plant rhizosphere interactions on phosphorus cycling and the significant differences in phosphorus cycling regulation mechanisms across different plant clusters and locations [44,45].

Alpine grasslands are located in globally sensitive climatic regions, where nutrient cycling is shaped not only by grazing but also by freeze–thaw processes, drought, and warming. Consequently, grazing regimes that alter nitrogen and phosphorus cycling pathways can further influence the resilience of these grasslands to future climate change. Our study demonstrates that mixed grazing significantly enhances nitrogen assimilation and phosphorus activation capacity. By strengthening endogenous nutrient cycling, mixed grazing helps buffer nutrient limitations under climatic warming, thereby supporting vegetation productivity and maintaining the stability of microbial functions. In contrast, the increased nitrogen losses associated with single-species grazing may undermine the grassland’s ability to cope with drought and temperature fluctuations. These findings indicate that scientifically informed grazing management has the potential to improve the climate resilience of alpine ecosystems.

#### 4.2.3. Intrinsic Regulatory Mechanisms of Soil N and P Cycling

The results of the Composite Variable Structural Equation Model indicate that grazing methods and plant clusters jointly influence the nitrogen and phosphorus cycling processes in alpine grassland soils through both direct and indirect pathways, with significant cluster-specific differences in the pathways and directions of these effects. The fertile island effect is commonly observed in grassland ecosystems under long-term grazing [15], and its intensity typically varies with plant species [17]. In *Carex alatauensis* clusters, grazing and the cluster exert opposing direct effects on TN, with grazing having a significant impact on nitrogen accumulation, while the cluster promotes nitrogen retention. Both grazing and the cluster have a negative impact on NO_3_^−^-N, suggesting that grazing disturbance may reduce nitrate nitrogen accumulation by enhancing plant absorption or limiting the supply of denitrification substrates [4]. The cluster has significant positive direct and indirect effects on NH_4_^+^-N, indicating its key role in nitrogen transformation processes. The rhizosphere microenvironment promotes ammonium nitrogen retention by inhibiting organic nitrogen mineralization and denitrification processes [46]. *Potentilla acaulis* clusters exhibited a different regulatory pattern. Grazing had a significant positive effect on TN, a negative effect on nitrate nitrogen, while the cluster indirectly promoted the accumulation of TN and nitrate nitrogen through nitrogen assimilation and denitrification processes. This suggests that nitrogen cycling in this cluster is primarily driven by plant rhizosphere processes, and grazing effects are mostly mediated through indirect pathways [47]. Grazing had no significant effect on ammonium nitrogen, reflecting the strong regulatory ability of *Potentilla acaulis* root systems on nitrogen forms.

In phosphorus cycling, *Carex alatauensis* clusters only exhibited a positive direct effect of the cluster on AP, indicating that its phosphorus cycling is mainly controlled by abiotic processes. The fertile island effect has a significant influence on the soil phosphorus cycling in *Carex alatauensis* clusters [17]. In contrast, grazing in *Potentilla acaulis* clusters promoted an increase in available phosphorus, while the cluster enhanced phosphorus recycling through organic phosphorus mineralization and uptake/transport processes [43]. Overall, grazing affects nitrogen and phosphorus availability by altering nutrient inputs and disturbance patterns [11,41], while plant clusters optimize nutrient cycling through the differential regulation of microbial functional genes in the clusters [1,18].

Against the backdrop of accelerating grassland degradation and nutrient depletion worldwide, the advantages of mixed grazing revealed in this study provide new ecological evidence for the sustainable management of grasslands at the global scale. By enhancing microbial functional diversity and improving both the efficiency and stability of nitrogen and phosphorus cycling, mixed grazing exemplifies the concept that “multi-species grazing optimizes ecosystem multifunctionality.” As a climate-sensitive region, the Qinghai–Tibetan Plateau offers a valuable case in which optimized grazing strategies not only hold regional significance but also provide important guidance for the management of cold-region grasslands globally.

## 5. Conclusions

This study elucidates the multilayered regulatory mechanisms of soil nitrogen and phosphorus cycling in alpine grasslands from the perspective of the combined effects of grazing regimes and plant clusters, highlighting the following key points: (1) dominant plant clusters form “fertile island effects” of differing intensities due to variations in species composition and rhizosphere environments, resulting in distinct nitrogen and phosphorus cycling pathways under grazing disturbance; (2) MG significantly increases the abundance of nitrogen-cycling genes such as *ureC* and *gs*, as well as phosphorus-related genes including *aphA*, *ugpB*, and *phnW*, thereby enhancing nitrogen assimilation and phosphorus activation and outperforming single-species grazing, with MG showing the strongest overall ecological benefits; (3) microbial functional genes respond to grazing disturbance more rapidly than changes in soil nutrient pools, underscoring their sensitivity as early indicators of ecosystem responses; (4) differences in rhizosphere regulation between *Potentilla anserina* and *Carex alatauensis* determine their nutrient-cycling characteristics—*Potentilla anserina* promotes nitrate and total nitrogen accumulation within internal clusters through enhanced nitrogen assimilation while external clusters experience increased denitrification and nitrate reduction, whereas *Carex alatauensis* maintains nitrogen stability by promoting ammonium accumulation; (5) structural equation modeling shows that grazing regimes and plant clusters jointly regulate nitrogen and phosphorus cycling through multiple direct and indirect pathways, demonstrating pronounced species specificity; and (6) MG optimizes plant–microbe interactions and nutrient redistribution, improving nutrient-use efficiency and functional stability in alpine grassland ecosystems, thus representing the most suitable management strategy for priority adoption in the Qinghai Lake region of the Qinghai–Tibetan Plateau.

## Figures and Tables

**Table 1:** Grazing experiment design details. In the table, YG represents yak-only grazing, SG represents sheep-only grazing, MG represents mixed grazing, and CK represents no grazing.

Treatment	Number of Yaks	Number of Tibetan Sheep	Area of Plot/m^2^	Grazing Intensity Sheep Units/ha	Number of Plots
YG	1	0	2.6 × 10^3^	3.86	3
SG	0	2	1.7 × 10^3^	3.86	3
MG	1	2	4.3 × 10^3^	3.86	3
CK	0	0	0.5 × 10^3^	0	3

## Data Availability

The original contributions presented in this study are included in this article. Further inquiries can be directed to the corresponding author.
